# Ambroxol reverses tau and α-synuclein accumulation in a cholinergic N370S *GBA1* mutation model

**DOI:** 10.1093/hmg/ddac038

**Published:** 2022-02-18

**Authors:** Shi Yu Yang, Jan-Willem Taanman, Matthew Gegg, Anthony H V Schapira

**Affiliations:** Department of Clinical and Movement Neurosciences, Queen Square Institute of Neurology, University College London, Royal Free Campus, London NW3 2PF, UK; Department of Clinical and Movement Neurosciences, Queen Square Institute of Neurology, University College London, Royal Free Campus, London NW3 2PF, UK; Department of Clinical and Movement Neurosciences, Queen Square Institute of Neurology, University College London, Royal Free Campus, London NW3 2PF, UK; Department of Clinical and Movement Neurosciences, Queen Square Institute of Neurology, University College London, Royal Free Campus, London NW3 2PF, UK

## Abstract

Cognitive impairment is a common non-motor complication of Parkinson’s disease (PD). Glucocerebrosidase gene (*GBA1*) variants are found in 10–15% of PD cases and are numerically the most important risk factor for PD and dementia with Lewy bodies. Accumulation of α-synuclein and tau pathology is thought to underlie cognitive impairment in PD and likely involves cholinergic as well as dopaminergic neurons. Neural crest stem cells were isolated from both PD patients with the common heterozygous N370S *GBA1* mutation and normal subjects without *GBA1* mutations. The stem cells were used to generate a cholinergic neuronal cell model. The effects of the *GBA1* variant on glucocerebrosidase (GCase) protein and activity, and cathepsin D, tau and α-synuclein protein levels in cholinergic neurons were examined. Ambroxol, a GCase chaperone, was used to investigate whether GCase enhancement was able to reverse the effects of the *GBA1* variant on cholinergic neurons. Significant reductions in GCase protein and activity, as well as in cathepsin D levels, were found in *GBA1* mutant (N370S/WT) cholinergic neurons. Both tau and α-synuclein levels were significantly increased in *GBA1* mutant (N370S/WT) cholinergic neurons. Ambroxol significantly enhanced GCase activity and decreased both tau and α-synuclein levels in cholinergic neurons. *GBA1* mutations interfere with the metabolism of α-synuclein and tau proteins and induce higher levels of α-synuclein and tau proteins in cholinergic neurons. The GCase pathway provides a potential therapeutic target for neurodegenerative disorders related to pathological α-synuclein or tau accumulation.

## Introduction

Although the loss of substantia nigra dopaminergic neurons is responsible for the dominant early motor features in Parkinson’s disease (PD), multiple neurotransmitter systems are known to be involved in this disorder. In the temporal staging of PD pathology proposed by Braak, Lewy bodies and neuronal loss in the substantia nigra occur concurrently with accumulation of α-synuclein (α-syn) deposition in cholinergic neurons of the basal forebrain neurons (1) suggesting that cholinergic denervation occurs early in PD. Both dopaminergic and cholinergic degeneration are likely to contribute to cognitive impairment in PD (2,3).

Glucocerebrosidase (GCase) is a lysosomal enzyme involved in the metabolism of glucosylceramide and is encoded by the *GBA1* gene. Homozygote *GBA1* mutations cause Gaucher’s disease, a systemic lysosomal storage disorder with a variable degree of involvement of the central nervous system. *GBA1* mutations (heterozygote and homozygote) are numerically the most important risk factor for the development of PD and for Dementia with Lewy bodies (DLB) (4,5). Cognitive impairment is reported to occur earlier and progress more rapidly in PD subjects with *GBA1* variants, including the N370S variant (6). Most *GBA1* variants reduce GCase activity and this in turn is associated with elevated levels of α-syn (7). We have previously reported on the biochemical consequences of the N370S *GBA1* variant in dopaminergic neurons derived from patients with PD (8). For the first time, we report the effects of the common N370S PD-associated *GBA1* variant in stem cell–derived cholinergic neurons to provide further insight into the potential mechanisms of cognitive dysfunction in *GBA1*-linked PD.

Cholinergic neuronal cell models have been generated from Alzheimer’s disease patients (9–11). Although different procedures and growth factors were used in the protocols, there was a common step in all these procedures, which is turning stem cells into neurospheres and then inducing neurospheres into cholinergic neurons. Neural crest stem cells (NCSCs) derived from adipose tissue have all the properties to form neurospheres (8), suggesting NCSCs may act as an alternative cell resource for generation of cholinergic neuronal models.

Tau is a member of microtubule-associated protein family and is involved in several neurodegenerative diseases. Tau pathology in neurodegenerative diseases is characterized by pathological tau aggregation in neurofibrillary tangles. The aggregation and deposition of tau were observed in approximately 50% of PD brains. Ambroxol (ABX) has been used for several years for the treatment of airway mucus hypersecretion and hyaline membrane disease in newborn babies. A drug screen then identified ABX as small molecule chaperone of GCase (12). Treatment of fibroblasts containing *GBA1* mutations with ABX resulted in increased level of GCase protein and its activity (13) and ABX has also been shown to increase GCase activity to reduce GCase substrate in macrophages with *GBA1* mutations (14). We employed ambroxol as a GCase enhancement agent to treat *GBA1* mutant (N370S) cholinergic neurons to examine whether increased GCase protein and activity affect the metabolism of α-syn and tau proteins in cholinergic neurons.

## Results

### Formation and characterization of neurospheres

**Figure 1:**
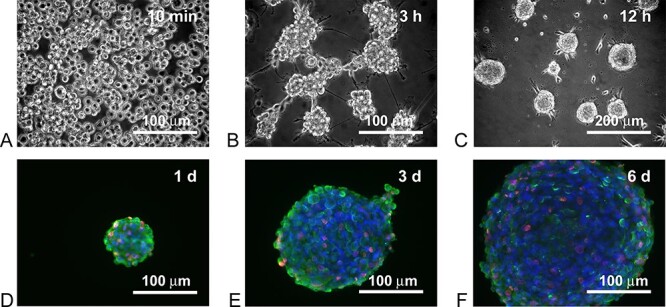
Human NCSCs were suspended in neurosphere formation medium for 10 min (**A**), after 3 h of incubation in neurosphere formation medium, NCSCs started to clump together (**B**). After 12 h of incubation in neurosphere formation medium, neurospheres were formed (**C**). BrdU incorporation assay indicated that the NCSCs continue proliferation in neurospheres over a 3-day period (**D**-**F**). Incorporated BrdU was immunostained fluorescent red, β-III tubulin was immunostained fluorescent green and nuclei were stained fluorescent blue with DAPI.

Adipose-derived NCSCs were converted to neurospheres as previously described (8) (Fig. 1A–C). A bromodeoxyuridine (BrdU) incorporation assay showed that 6 days after conversion cells were still proliferating, as also reflected by the increase in size of the neurospheres (Fig. 1D–F).

**Figure 2:**
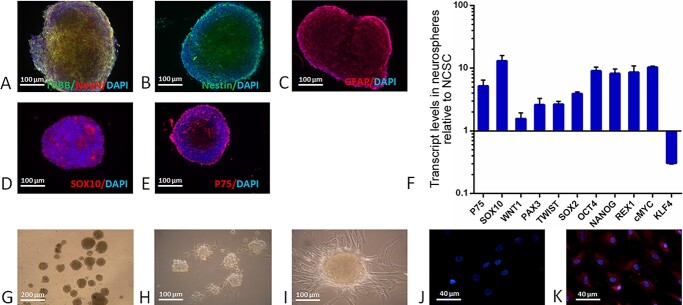
The neural markers β-III tubulin (**A**), NeuN (A) and Nestin (**B**), the astrocyte marker GFAP (**C**), and the NCSC markers SOX10 (**D**) and P75 (**E**) were expressed in neurospheres. The transcript levels of the NCSC markers P75 and *SOX10*, and the pluripotent genes *WNT1*, *PAX3*, *TWIST*, *SOX2*, *OCT4*, *NANOG*, *REX1* and *cMYC* were increased in neurospheres compared with NCSCs, whereas transcript level of the transcription factor *KIF4*, which mediates migration, was decreased (**F**). Neurospheres were formed and maintained in suspension in neurosphere formation medium (**G**). Neurospheres attached to the surface of a well in a fibronectin-coated plate (**H**). Medial ganglionic eminence (MGE) cells formed in pre-cholinergic neuronal differentiation medium (**I**). Higher level of NKX2-1 expression (red) occurred after 7 days of incubation in pre-cholinergic neuronal differentiation medium (**K**) as compared with 5 days incubation (**J**).

The neurospheres were characterized by immunocytochemistry and RT-PCR (Fig. 2). The results showed that the cells expressed typical neuronal markers, such as β-III tubulin (Fig. 2A), NeuN (Fig. 2A) and Nestin (Fig. 2B), the astrocyte marker GFAP (Fig. 2C), and NCSC markers SOX10 (Fig. 2D) and P75 (Fig. 2E). The transcript levels of the pluripotent genes *WNT1*, *PAX3*, *TWIST*, *SOX2*, *OCT4*, *NANOG*, *REX1* and *cMYC* increased 2–10 times in neurospheres compared with NCSCs (Fig. 2F). NCSCs are highly migratory cells (15). The *KLF4* gene encodes a transcription factor mediating cellular migration (16). As NCSCs form neurospheres and lose their migration ability, the expression of *KLF4* in neurospheres decreased compared with NCSCs (Fig. 2F). These data indicate that NCSC-derived neurospheres have similar characteristics as embryonic stem cell or induced pluripotent stem cell (iPSC)-derived neurospheres, which have been used to generate functional basal forebrain cholinergic neurons (17,18).

### Induction of neurospheres to medial ganglionic eminence cells

The first step for differentiation of neurospheres to cholinergic neurons is to induce transition to medial ganglionic eminence (MGE) cells (19). A fibronectin-coated plate was used to make suspended neurospheres (Fig. 2G) attach to the plate surface (Fig. 2H). Cells were further incubated with neurobasal medium supplemented with B27, FGF2 and leukaemia inhibitory factor for up to7 days. MGE cells were formed and migrated from the neurospheres (Fig. 2I). The homeobox protein NKX2–1 is an MGE cell marker (19). A higher level of NKX2–1 expression was seen at 7 days of incubation (Fig. 2K) compared with 5 days (Fig. 2J).

### Characterization of cholinergic neurons

**Figure 3:**
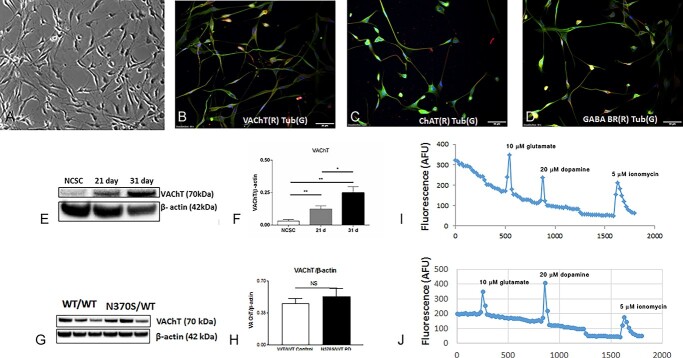
Morphology of cholinergic neurons after 31 days of differentiation (**A**). Neuronal marker β-III tubulin (green) was expressed in most of the cells (**B**, **C** and **D**). Cholinergic neuronal markers VAChT (red) (B), ChAT (red) (C) and the GABA receptor (red) (D) were expressed in differentiated cells. The expression level of VAChT increased during the differentiation (**E** and **F**). VAChT levels were compared between normal (WT/WT) and *GBA1* mutant (N370S/WT) cholinergic neuronal cultures (**G** and **H**). Single-cell transient cytoplasmic Ca^2+^ fluxes of NCSC-derived cholinergic neurons upon application of 10 μM glutamate, 20 μM dopamine and 5 μM ionomycin in normal (WT/WT) (**I**) and *GBA1* mutant (N370S/WT) cultures (**J**).

After 31 days of cholinergic neuronal differentiation, most cells showed neuronal cell morphology (Fig. 3A). The vesicular acetylcholine transporter (VAChT) is a neurotransmitter transporter responsible for transferring acetylcholine into secretory vesicles (20). VAChT is regarded as a specific marker for cholinergic neurons and has been widely used for the study of cholinergic transmission in experimental models of Alzheimer’s disease and other disorders involving cholinergic neurons (21). Immunostaining showed that the neuronal marker β-III tubulin was expressed in the majority of the NCSC-derived cholinergic neurons after 31 days of differentiation (Fig. 3B, C and D, and Supplementary Material, Fig. S1), whereas VAChT was expressed in 45–60% of the cells (Fig. 3B and Supplementary Material, Fig. S1). Immunoblotting demonstrated that VAChT levels progressively increased during the 31-day differentiation protocol (Fig. 3E and F). Choline acetyltransferase (ChAT) plays a key role in the biosynthesis of the neurotransmitter acetylcholine and is a marker for cholinergic neurons. GABA-B receptors are located on nerve terminals of cholinergic neurons and mediate inhibition of nerve-stimulated release of acetylcholine. Both ChAT and GABA-B receptors have been used to identify cholinergic neurons in previous studies (22,23). Immunostaining revealed that 35–45% of the differentiated cells expressed ChAT and GABA-B receptors, while > 85% expressed the neuronal marker β-III tubulin (Fig. 3C and D, and Supplementary Material, Fig. S1).

To investigate if the N370S *GBA1* variant affects the function of ligand-gated Ca^2+^ channels in the NCSC-derived cholinergic neurons, we measured intracellular Ca^2+^ changes in neurons loaded with the Ca^2+^ indicator dye Fluo-4 AM in response to different neurotransmitters. A similar approach has been used to analyse human embryonic stem cell–derived cholinergic neurons (18). The receptors for glutamate and dopamine have previously been demonstrated in cholinergic neurons (24,25). Our NCSC-derived cholinergic neurons were stimulated with glutamate and dopamine. Towards the end of the recordings, the ionophore ionomycin was added to the medium to confirm the viability of the cells. The cells were responsive to both glutamate and dopamine. We observed no difference between the transient cytosolic Ca^2+^ changes in the *GBA1* variant (N370S/WT) and healthy (WT/WT) NCSC-derived cholinergic cells (Fig. 3I and J). To examine whether the N370S variant affects cholinergic neuronal differentiation, we compared VAChT expression levels between N370S/WT and control NCSC-derived cholinergic neurons (Fig. 3G and H). The level of VAChT in both neuronal types was not significantly different, indicating the *GBA1* variant (N370S/WT) did not affect neuronal differentiation.

### Increased levels of tau and α-syn in N370S *GBA1* variant cholinergic neurons

**Figure 4:**
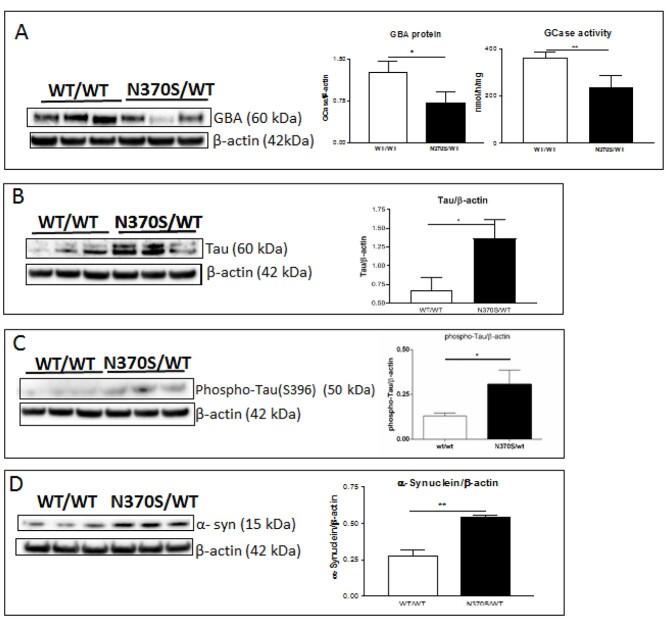
GCase protein (GBA) and activity in normal (WT/WT) and *GBA1* mutant (N370S/WT) cholinergic neurons (**A**). Tau protein in control (WT/WT) and *GBA1* mutant (N370S/WT) cholinergic neurons (**B**). Phospho-tau (S396) protein in control (WT/WT) and *GBA1* mutant (N370S/WT) cholinergic neurons (**C**). α-syn protein in normal (WT/WT) and *GBA1* mutant (N370S/WT) cholinergic neurons (**D**).

Microtubule-associated protein tau (encoded by the *MAPT* gene) is expressed mainly in neurons of the central nervous system and is a regulator of tubulin assembly in neuronal cells (26). Mutations in *MAPT* cause neurodegenerative tauopathies, e.g. progressive supranuclear palsy and frontotemporal dementia. Aggregated tau is also found in other neurodegenerative disorders, such as Alzheimer’s disease and DLB. Tau and α-syn inclusions are present in cholinergic neurons of synucleinopathies associated with dementia (27). Increased tau phosphorylation at Ser396 (S396) along with α-syn has been reported in synapse-enriched fractions from PD brains. The co-occurrence of phosphorylated species of tau and α-syn and the presence of both in Lewy bodies suggest a possible physiological or pathophysiological interaction (28). We examined the levels of tau, phospho-tau (S396) and α-syn proteins in control and N370S/WT *GBA1* variant cholinergic neurons. Compared with controls, GCase protein level and enzyme activity were significantly decreased, respectively 41% and 32% lower in N370S/WT cholinergic neurons (Fig. 4A). GCase protein and activity decreased in parallel suggesting that the low GCase activity is due to a lower level of enzyme protein. Compared with controls, tau and phospho-tau (S396) levels were significantly increased, respectively 164% and 120% higher in N370S/WT cholinergic neurons (Fig. 4B and C), while α-syn levels were also significantly increased by 105% in N370S/WT cholinergic neurons (Fig. 4D).

### Effects of the *GBA1* N370S mutation on cathepsin D and macroautophagy pathways in cholinergic neurons

**Figure 5:**
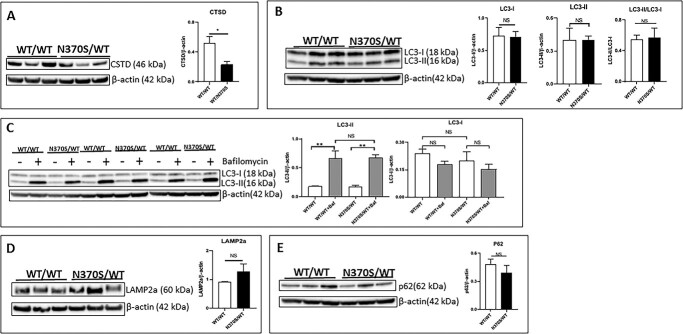
Cathepsin D (CTSD) protein in control (WT/WT) and *GBA1* mutant (N370S/WT) cholinergic neuronal cultures (**A**). LC3-I and LC3-II protein in control (WT/WT) and *GBA1* mutant (N370S/WT) cholinergic neuronal cultures (**B**). LC3-I and LC3-II protein in control (WT/WT) and *GBA1* mutant (N370S/WT) cholinergic neuronal cultures treated with vehicle (−) or 100 nM bafilomycin (+) for 6 h (**C**). LAMP-2a protein in control (WT/WT) and *GBA1* mutant (N370S/WT) cholinergic neuronal cultures (**D**). p62 protein in control (WT/WT) and *GBA1* mutant (N370S/WT) cholinergic neuronal cultures (**E**).

Our previous study reported that the N370S/WT *GBA1* variant reduced cathepsin D (CTSD) protein and activity in dopaminergic neuronal cells (29). We therefore compared CTSD protein levels between control and N370S/WT cholinergic neurons. CTSD levels were significantly lower in N370S/WT cholinergic neurons compared with control (Fig. 5A).

Tau and α-syn turn over through the autophagy pathway. Therefore, we examined whether GCase dysfunction had any effects on macroautophagy in cholinergic neurons. The macroautophagic flux can be evaluated by assessing the levels of post-translationally modified microtubule-associated protein 1A/1B-light chain 3 (LC3). Cytosolic LC3 is cleaved to form LC3-I, which is subsequently conjugated to phosphatidylethanolamine to form LC3-II and recruited to the autophagosomal membranes. We measured the levels of LC-I and LC-II in control and N370S/WT cholinergic neurons on immunoblots. Calculation of the LC3-II/LC3-I ratios revealed no significant differences, indicating that basal macroautophagy is not affected by the *GBA1* (N370S/WT) genotype (Fig. 5B). To determine if the *GBA1* (N370S/WT) variant changes the flux of LC-II through the macroautophagy pathway, we measured LC-II levels in cultures treated with the lysosomal inhibitor bafilomycin. As expected, bafilomycin treatment resulted in an increase of LC3-II levels because it inhibits lysosomal LC3-II degradation. Bafilomycin-treated control and N370S/WT cholinergic neurons showed no difference in LC3-II levels (Fig. 5C). This implies that the macroautophagic flux is not affected by the *GBA1* (N370S/WT) genotype.

The notion that the *GBA1* (N370S/WT) genotype does not affect the macroautophagy pathway in cholinergic neuronal cells was further supported by immunoblots probed for LAMP-2A and p62. The lysosomal protein LAMP-2A is an important component of chaperone-mediated autophagy in neurons. LAMP-2A levels of N370S/WT and control neurons showed no significant differences on immunoblots (Fig. 5D). Likewise, levels of the autophagy receptor protein p62, encoded by the *SQSTM1* gene, were not significantly different in the control and N370S/WT cholinergic cultures (Fig. 5E).

### Ambroxol (ABX) treatment increases GCase protein level and activity

**Figure 6:**
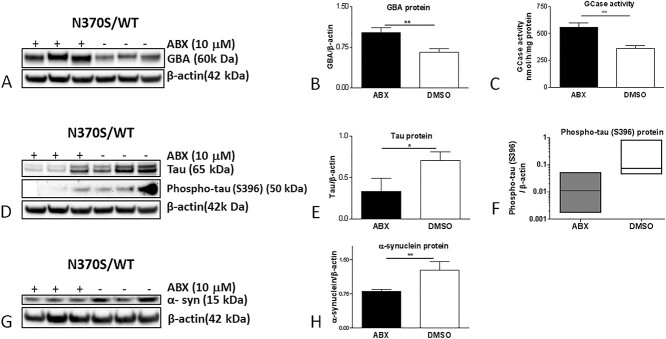
GCase protein (GBA) and activity in ABX-treated and untreated *GBA1* mutant (N370S/WT) cholinergic neurons (**A**, **B** and **C**). Tau and phospho-tau (S396) proteins in ABX-treated and untreated *GBA1* mutant (N370S/WT) cholinergic neurons (**D**, **E** and **F**). Monomeric α-syn protein in ABX-treated and untreated *GBA1* mutant (N370S/WT) cholinergic neurons (**G** and **H**). To highlight the variability in phospho-tau levels between the different cultures, a box plot indicating median ± SEM was used in panel F.

ABX is a GCase pharmacological chaperone and has been reported to increase the expression of the TFEB transcription factor (30) and increase GCase protein and activity in human dopaminergic neurons (8). N370S/WT *GBA1* variant cholinergic neurons were treated with ABX for 6 days. The treatment resulted in significant increases of GCase protein level and activity by 50% and 55%, respectively (Fig. 6A, B and C). ABX treatment significantly reduced tau levels to 44% of basal levels (Fig. 6D and E). Phospho-tau (S396) levels varied in the different vehicle-treated N370S/WT *GBA1* cholinergic cultures (Fig. 6D), but treatment with ABX resulted in an 8- to a 20-fold decrease of phospho-tau (S396) levels (Fig. 6D and F). Levels of α-syn were significantly decreased to 59% of basal levels following ABX treatment (Fig. 6G and H).

## Discussion

Cognitive impairment is a common feature of PD. *GBA1* sequence variants are found in 10–15% of PD patients and their presence is associated with earlier onset and more rapid progression of cognitive dysfunction (6,31–33). The cognitive decline seen in PD is thought to represent pathology in both dopaminergic and cholinergic neuronal pathways. To date, no study has yet investigated the potential effects of *GBA1* mutations on cholinergic function and whether the biochemical consequences of these might accelerate proteinopathy related to cognitive decline. In the present study, NCSCs from PD patients with the N370S *GBA1* variant were used to generate cholinergic neurons. We found that GCase enzyme activity, and GCase and CTSD protein levels were significantly reduced, and the levels of tau, phospho-tau (S396) and α-syn were significantly increased in N370S/WT cholinergic neurons. The ratio of LC3-II/LC3-I and autophagic flux were not affected by the *GBA1* variant, suggesting that the increase in tau and α-syn after 31 days differentiation is not mediated by impaired macroautophagy in this cholinergic cell model. Although the protein levels of LAMP2A were also unchanged, it cannot be excluded that its assembly into the multimeric assembly complex might affect degradation of these proteins via CMA (34).

CTSD-mediated proteolysis is essential for neuronal degradation of unfolded protein aggregates that reach the lysosomes via autophagy or endocytosis (35). Many neuronal proteins, such as α-syn (36) and tau (37), are physiological substrates of CTSD. The decreased CTSD protein in the N370S *GBA1* mutant cholinergic neurons may explain the increased levels of these proteins in the mutant neurons. Decreased CTSD activity has been found in brains of PD and dementia with Lewy body patients with and without *GBA1* variants and correlated with both reduced GCase activity and *GBA1* gene expression (38). We have previously published the effects of the N370S *GBA1* variant on dopaminergic neurons and found GCase protein and activity significantly decreased (8), with a significant reduction of CTSD protein and activity, and an increase in α-syn levels (29). Reduced expression of CTSD has also been reported in *GBA1*-PD (39). The mechanism for why mature CTSD expression is reduced in *GBA1*-PD neurons is unclear. Cathepsins are expressed as pro-proteins, maturing as they reach the acidified environment of endolysosomes. Loss of GCase activity likely affects the lipid profile of cells that might influence the transport of proteins to lysosomes and/or the pH of lysosomes. The imbalance of both sphingolipids and phospholipids has also been implicated in the mishandling of α-syn (40). While we have not measured lipids in this study, ABX treatment has been reported to lower GCase substrate in macrophages treated with ABX (14) and might contribute to the reduction in at least α-syn in our models following chaperone treatment. In addition to autophagy, soluble α-synuclein and tau can be degraded by the ubiquitin proteasome system (UPS) (41). The UPS has been reported to be decreased in the brain of *Gba1* knockout mice (42) and might also contribute to the accumulation of a-syn and/or tau in this cholinergic model.

It has previously been suggested that both α-syn and tau proteins play an important role in the pathogenesis of cognitive dysfunction in PD (38). Our observation that N370S *GBA1* cholinergic cells have increased levels of these two proteins may help provide an explanation for the earlier onset and more rapid progression of dementia associated with this mutation (43). This is supported by the observation of both α-syn and tau aggregates in the hippocampus of a homozygous *Gba1* mutant mouse model, which were coincident with memory deficits (44). Notably, the increased tau we observe in our cholinergic cells was phosphorylated at the same residues as tau aggregates associated with the pathology of Alzheimer’s disease and tauopathies.

ABX has been shown to reverse the biochemical consequences of *GBA1* mutations, increasing GCase enzyme activity and reducing α-syn levels in a range of cell types, including human dopaminergic neurons with the N370S *GBA1* variant and in vivo models (8,30,45,46). ABX has most recently been used in the first clinical trial of a personalized medicine for PD (47). It has been shown to be brain penetrant and to increase GCase protein levels in cerebrospinal fluid in PD patients with and without *GBA1* mutations. ABX has been proposed as a potential disease-modifying drug to slow onset and progression in PD. The results of the present study are important in terms of the potential for ABX to reduce tau, phospho-tau and α-syn levels in cholinergic neurons and potentially influence the onset and progression of cognitive decline in PD. Its ability to reduce tau, phospho-tau and α-syn in cholinergic cells, as well as α-syn in dopaminergic cells, could represent an important protective mechanism against dementia in PD, and *GBA1*-PD in particular.

## Materials and Methods

### Study design and participants

This study was approved by the Royal Free Research Ethics Committee, Royal Free Hospital, London, UK (REC number 10/H0720/21). Written consent was obtained from each subject included in the study, in accordance with the Declaration of Helsinki. Three PD patients (2 females, 1 male, ages 55, 75 and 80) carrying heterozygous *GBA1* mutations (WT/N370S) and three healthy controls (1 female, 2 males, ages 56, 59 and 73) confirmed to have no *GBA1* mutation, participated in the study. Demographic, neurological and cognitive data were collected. The control subjects had no neurological or cognitive deficits by history. DNA from all participants was studied by whole-exome analysis.

### Sample collection and cell isolation

Subcutaneous fat was collected by skin biopsy. The previous published procedures were followed for the sample preparation and NCSC isolation (8). The six individual subjects were divided into two groups according to their genotype (WT/WT healthy, N370S/WT PD).

### Growth factors

Recombinant human/mouse fibroblast growth factor 8b (FGF-8b), recombinant human fibroblast growth factor-2 (FGF-2, 146 amino acids), epidermal growth factor (EGF), recombinant human bone morphogenetic protein-9 (BMP-9), recombinant human/mouse/rat/canine/equine brain-derived neurotrophic factor (BDNF) and leukemia inhibitory factor (LIF) were purchased from R and D Systems.

### Culture media

Stem cell growth medium consisted of Dulbecco’s modified Eagle medium (DMEM; high glucose, GlutaMAX, Life technologies) supplemented with fetal bovine serum (10%), sodium pyruvate (1 mM), uridine (50 μg/ml), penicillin (50 units/ml), streptomycin (50 μg/ml) and amphotericin B (1.25 μg/ml). Neurosphere formation medium consisted of DMEM/F12 medium supplemented with B27 (1×), FGF-2 (20 ng/ml), EGF (10 ng/ml), LIF (10 ng/ml), penicillin (50 units/ml), streptomycin (50 μg/ml) and amphotericin B (1.25 μg/ml). Pre-cholinergic neuronal differentiation medium consisted of DMEM/F12 medium supplemented with B27 (1×), FGF-2 (20 ng/ml), LIF (10 ng/ml), BMP-9 (10 ng/ml), penicillin (50 units/ml), streptomycin (50 μg/ml) and amphotericin B (1.25 μg/ml). Cholinergic neuronal differentiation medium consisted of DMEM/F12 medium supplemented with B27 (1×), FGF-2 (20 ng/ml), LIF (10 ng/ml), BMP-9 (10 ng/ml), BDNF (20 ng/ml), penicillin (50 units/ml), streptomycin (50 μg/ml) and amphotericin B (1.25 μg/ml).

### Neurosphere formation and cholinergic neuronal differentiation

NCSCs were cultured as described previously (8), harvested in stem cell growth medium and centrifuged at 200×*g* to collect cell pellets. Cell pellets were re-suspended in neurosphere formation medium and plated in non-coated (low adhesion) culture dishes. For cholinergic neuronal differentiation, neurospheres were transferred to a fibronectin-coated 6-well plate with neurosphere formation medium. Following 24-h culturing, during which neurospheres attached to the surface of plate, medium was replaced with fresh neurosphere formation medium. The next procedure was divided into 3 stages. Stage 1: neurospheres were cultured in neurosphere formation medium for a further 6 days, medium was changed on day 3, 1 mL of medium was removed and 2 mL of freshly made medium was added to each well during medium change. Stage 2: neurosphere formation medium was removed from each well, and 2 mL of fresh pre-cholinergic neuronal differentiation medium was added to each well. Cells were cultured in pre-cholinergic neuronal differentiation medium for 12 days; medium was changed every 4 days; 1 mL of medium was removed and 2 mL of fresh medium was added to each well during medium change. Stage 3: pre-cholinergic neuronal differentiation medium was removed from each well, and 2 mL of fresh cholinergic neuronal differentiation medium was added to each well. Cells were cultured in cholinergic neuronal differentiation medium for 12 days; medium was changed every 4 days; 1 mL of medium was removed and 2 mL of fresh medium was added to each well during medium change.

### ABX treatment

Medium was removed; fresh cholinergic neuronal differentiation medium supplemented with 10 μM ABX was added to each well (2 mL/well, 6-well plate); medium was changed every 48 h. Treatment lasted for 6 days. Control cells were treated with vehicle (dimethyl sulfoxide, DMSO) instead of ABX.

### GCase enzyme activity assays

Cell pellets were lysed with 1% Triton X-100 in PBS. GCase activity was determined in cell lysates of ~1 μg protein as previously reported (7). Enzyme activities were calculated by subtracting the background fluorescence from the mean fluorescence measured for a given cell lysate and then divided by the standard to calculate the activity in nmol/h/ml. This result was then divided by the total protein concentration, as determined using bicinchoninic acid assay method, to calculate the enzymatic activity in nmol/h/mg.

### Immunochemistry

Cells were washed twice with phosphate-buffered saline (PBS), each wash lasting 5 min. Cells were fixed with 4% paraformaldehyde in PBS for 15 min at room temperature and subsequently permeabilized with 0.25% Triton X-100 in PBS for 15 min. Following three PBS washes, cells were blocked with 10% goat serum in PBS for 30 min and incubated with primary antibodies (Supplementary Material, Table SS1) overnight at 4°C. The appropriate secondary antibodies conjugated with Alexa Fluor-448 or Alexa Fluor-568 (Invitrogen) were used to visualize the immunoreactive cells. Nuclei were stained with DAPI.

### Immunoblotting

Cells were harvested, washed with PBS and processed as previously described (8). Primary antibodies are given in Supplementary Material, Table S1.

### BrdU incorporation assay

Neurospheres floating on glass coverslips were cultured with 10 μM 5-bromo-2′-deoxyuridine (BrdU) for 4 h. Detection of incorporated BrdU was carried out with the 5-bromo-2′-deoxyuridine labelling and detection Kit I (Roche/Sigma-Aldrich) as recommended by the supplier. Co-staining with additional primary antibodies (Supplementary Material, Table S1) was performed during the anti-BrdU antibody incubation step. Samples were incubated with appropriate Alexa Fluor 488- and Alexa Fluor 594-conjugated secondary antibodies and further processed for immunocytochemistry.

### Intracellular Ca ^2+^ imaging

To evaluate transient intracellular Ca^2+^ concentrations, cells cultured in 35-mm μ-dishes with a glass bottom (Ibidi) were loaded for 30 min at room temperature with 5 μM Fluo-4 AM (Thermo Fisher Scientific) and 0.02% pluronic acid in Krebs-Ringer modified buffer composed of 135 mM NaCl, 5 mM KCl, 2 mM MgSO_4_, 1.25 mM KH_2_PO_4_, 1 mM CaCl_2_, 10 mM glucose and 10 mM HEPES (pH 7.4). Prior to fluorescence measurements, cells were washed and incubated in indicator-free medium for a further 30 min. Fluorescent signals were recorded with a Nikon Eclipse Ti-E inverted confocal laser-scanning microscope, equipped with a × 10 objective. Cells were stimulated with 10 μM l-glutamate, 20 μM dopamine and 5 μM ionomycin. Imaging data were collected with NIS-Elements software (Nikon). For the measurements, single cells were selected with Image J software (National Institutes of Health) and the green fluorescent signal was plotted against time.

### Statistical analysis

Data are expressed as mean ± SEM. Statistical significance between groups was determined by one-way ANOVA followed by a two-tailed *t*-test. A *P-*value of <0.05 was considered as significantly different. All data were analysed by GraphPad Prism 6 statistical software.

*Conflict of Interest statement.* None.

## Funding

JPND through the MRC (grant code MR/T046007/1) and Parkinson’s UK (grant G-1704). A.H.V.S. is supported by the UCLH NIH BRC.

## Authors’ Contributions

S.Y.Y. and A.H.V.S. designed the research; S.Y.Y. and J.-W.T. performed the experiments; all authors participated in data analysis; all authors participated in manuscript drafting and revision. A.H.V.S. supervised the project.

## Supplementary Material

Suppl_Fig_1_ddac038

Suppl_Table_1_ddac038
