# Supplementary material for: Ambroxol reverses tau and α-synuclein accumulation in a cholinergic N370S GBA1 mutation model
Source: Hum Mol Genet. 2022 Feb 18;31(14):2396–405. doi: 10.1093/hmg/ddac038 (PMC9307316; doi:10.1093/hmg/ddac038)
Supplement: Suppl_Fig_1_ddac038 [file suppl_fig_1_ddac038.pdf]

Supplementary Figure 1

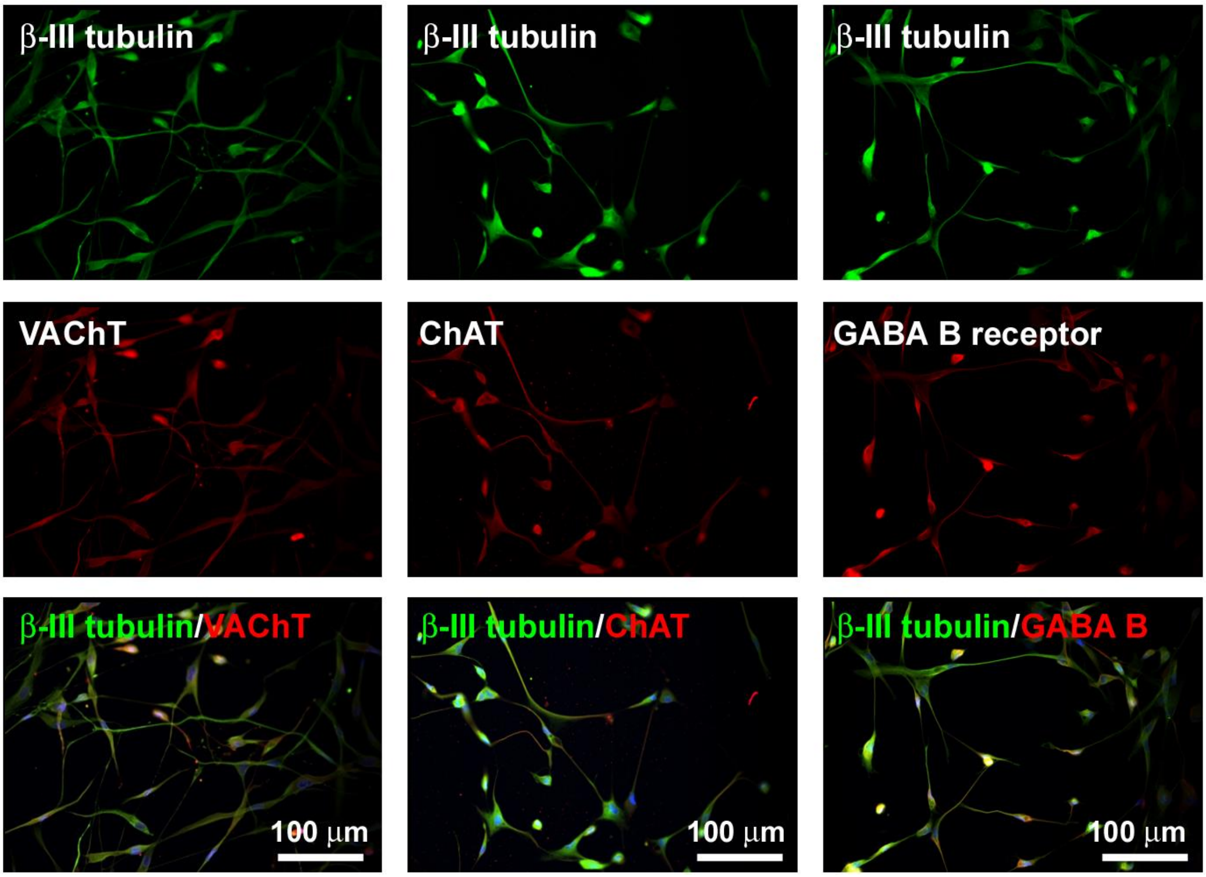

Supplementary Figure 1 Expression of marker proteins in cholinergic neurons after 31 days of differentiation. The neuronal marker β-III tubulin (green) was expressed in most cells. The cholinergic neuronal markers VAChT (red) and ChAT (red), and the GABA B receptor (red) were expressed in the majority of differentiated cells.
