# Supplementary material for: Ambroxol reverses tau and α-synuclein accumulation in a cholinergic N370S GBA1 mutation model
Source: Hum Mol Genet. 2022 Feb 18;31(14):2396–405. doi: 10.1093/hmg/ddac038 (PMC9307316; doi:10.1093/hmg/ddac038)
Supplement: Suppl_Table_1_ddac038 [file suppl_table_1_ddac038.pdf]

Supplementary Table 1 List of primary antibodies.

Antibody	Dilution	Application	Source	Cat. No.
α-synuclein	1:500	WB	Abcam	Ab1903
β-III tubulin	1:500	ICC	Abcam	Ab18207
ChAT	1:200	ICC	Abcam	Ab2803
CTSD	1:1000	WB	Abcam	Ab134169<br>Ab75852
GABA receptor	1:200	ICC	Abcam	Ab55051
GBA1	1:1500	WB	Calbiochem	AP1140
GFAP	1:500	ICC	Millipore	Mab360
LAMP2a	1:1000	WB	Abcam	Ab18528
LC3-I and LC3-II	1:1000	WB	Cell signalling	2775
Nestin	1:500	ICC	Millipore	Mab5326
Neu N	1:500	ICC	Millipore	ABN78
P62	1:1500	WB	BD Biosciences	610832
P75 NGF R	1:500	ICC	Abcam	Ab8874
Phospho-tau	1:300	WB	Abcam	Ab109390
SOX 10	1:250	ICC	Santa Cruz	Sc17342
Tau	1:1000	WB	MERCK	MAB2241
VAChT	1:1000	ICC	Sigma	SAB4200560
